# The Effect of Single Recombination Events on Coalescent Tree Height and Shape

**DOI:** 10.1371/journal.pone.0060123

**Published:** 2013-04-08

**Authors:** Luca Ferretti, Filippo Disanto, Thomas Wiehe

**Affiliations:** 1 Institut für Genetik, Universität zu Köln, Köln, Germany; 2 Center for Research in AgriGenomics, Barcelona, Spain; North Carolina State University, United States of America

## Abstract

The coalescent with recombination is a fundamental model to describe the genealogical history of DNA sequence samples from recombining organisms. Considering recombination as a process which acts along genomes and which creates sequence segments with shared ancestry, we study the influence of single recombination events upon tree characteristics of the coalescent. We focus on properties such as tree height and tree balance and quantify analytically the changes in these quantities incurred by recombination in terms of probability distributions. We find that changes in tree topology are often relatively mild under conditions of neutral evolution, while changes in tree height are on average quite large. Our results add to a quantitative understanding of the spatial coalescent and provide the neutral reference to which the impact by other evolutionary scenarios, for instance tree distortion by selective sweeps, can be compared.

## Introduction

Coalescent theory is a central part of modern population genetics [1]–[3]. It constitutes the basis of genealogical models, of statistical tests of the neutral evolution hypothesis [4] as well as of many simulation tools [5]–[7]. Besides application in population genetics, coalescent models and their various generalizations became an object of study in their own right in probability, graph theory and combinatorics [8]–[12].

**Figure 1:**
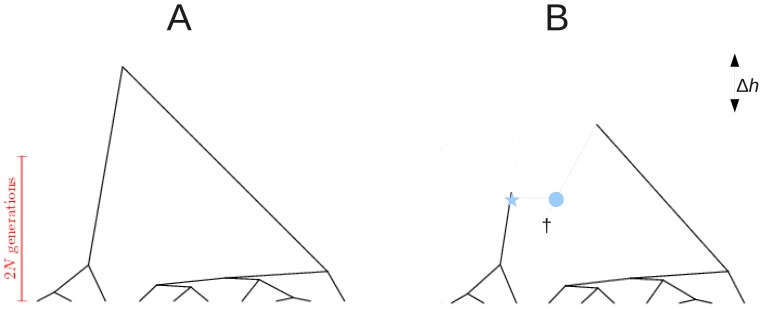
Example coalescent trees. A: Tree of size 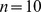 generated under the coalescent process. The 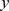-axis represents a time scale, with leafs at the ‘present’, and the root in the ‘past’. Starting from the present and going backwards in time, coalescent events are exponentially distributed with a parameter depending on population size (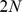) and the number of lineages at any given point in time. B: Recombination is a prune (asterisk) and re-graft (circle) event: a lineage splits and merges onto another lineage which exists in the population at the time of recombination. This lineage does not need to extend to the present, and it may have become extinct from the entire population (cross). Recombination has changed the height of the coalescent tree with respect to the tree in panel A (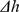), but has not changed root imbalance: for both trees 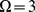.

The classical coalescent is a binary, rooted, unordered tree with a fixed number 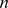 of leafs. The latter is also called the *size* of the tree (Figure 1A). Such a tree can be interpreted as the genealogical history of a sample of DNA sequences, where mergers (“coalescents”) of two lineages represent events of common ancestry. Thus, coalescent trees are naturally fitted with a time scale and for this reason they are sometimes called *labelled histories*. A biologically important generalization of the simple case is the coalescent with recombination. Recombination is a process by which two DNA sequences reciprocally exchange genetic material. In the coalescent framework this translates into lineage splits (Figure 1B). A split represents the un-coupling of the genealogical history of two sequence fragments. The ancestral recombination graph (ARG) [13] is a model to integrate such lineage splits into coalescent trees. Each sequence position 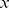 along the chromosome is associated with a coalescent tree 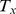, which is the marginal tree of the ARG at position 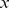. Depending on the rate of recombination, chromosomes are divided into smaller or larger sequence fragments 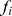 (“haplotype block”) in such a way that all positions within a fragment are free of recombination and therefore have the same marginal tree 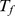.

The spatial coalescent is the sequence 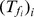 of coalescent trees along a sample of recombining chromosomes. Study of the spatial coalescent is of prominent interest in population genomics, since it contains information about the demographic and evolutionary history of a population. For instance, it has lately been used to infer demographic parameters in non-African human [14]. Unfortunately, the spatial coalescent is not a simple Markov process [15], complicating its probabilistic analysis and leaving many open problems to be addressed.

**Figure 2:**
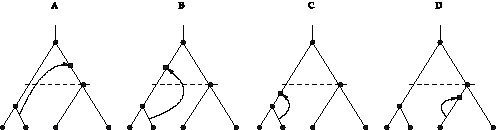
Non-silent, silent and hidden recombination events. A: Non-silent recombination changes tree topology. In the case shown, also 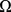 changes from 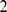 to 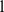. B: A recombination event which changes the order of internal nodes. Whether this event is classified as non-silent or silent, depends on the tree definition. It is non-silent for labelled histories (considered here; eq (1)), but it would be silent for unlabelled trees. C: A silent recombination event, which does not affect the branching pattern, but the lengths of the recombining branches. D: A hidden recombination event. It does neither affect branching pattern nor branch lengths.

Here, we investigate the impact of single recombination events upon some measures of tree topology and shape. By *topology* we mean the branching pattern of a tree; by *shape* we mean its topology and branch lengths. In particular, we ask how recombination affects tree height and tree (im-)balance. The latter is measured by the difference in size of the left and right subtrees emerging from the root or any internal node. Depending on when and where a recombination event occurs, the effect on altering tree structure may be drastic, mild or completely silent. Informally, drastic events are those which lead to a large change of tree height or balance. These are events which typically involve splits by recombination of the branches emerging from the root of the tree. As such they may strongly affect the genealogical structure of haplotypes. Identifying and characterizing these events is very informative for population genetic inference. Mild events are typically those which occur along very recent branches, close to the leafs of the tree. They do not, or only mildly, affect haplotype structure and mutation frequency spectrum. Interestingly, there is a non-negligible portion of recombination events which do not alter tree topology, i.e. the branching pattern. We call these events *silent*. Sometimes, also the branch lengths remain unchanged; we call these events *hidden* (Figure 2).

Our goals are to formalize these concepts, to characterize in more detail the effect of single recombination events upon tree shape and to quantify the relative frequencies of drastic, mild and silent events. We explicitly calculate the probabilities of changes in height or root balance induced by a single recombination event. Our results are based on the assumption of a standard neutral model of constant population size. This means that for each coalescent event two lineages are chosen at random to merge. Further, the timing of events is exponentially distributed with a rate which, after re-scaling by population size 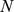, depends only on the number of lineages at a given time.

In Results Section (a), we define a probability density for the trees in the spatial coalescent and we explain the difference between pointwise marginal trees 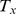, evaluated at every basepair 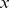 of the DNA sequences, and the marginal trees 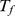, evaluated at every fragment 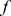. We derive a simple relation between the densities of 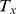 and 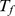. In Section (b) we analyze the recombination events which lead to height-changes and derive their probabilities. In Section (c) we quantify the concept of root imbalance, called 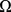, and derive the first-order transition probabilities under single recombination events. We focus on events which produce unbalanced trees and, at the same time, lead to an increase of tree height. This type of events is of particular interest for the analysis of biological data. Their effect on the mutation frequency spectrum and on haplotype structure is the basis of tests to reject the neutral evolution hypothesis (e.g., [16]–[18]). Therefore, for bench-marking it is highly interesting to know how often such events occur under purely neutral conditions, but it is not the goal of this paper to devise another neutrality test. Then, we generalize the results regarding the tree topology parameter 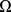 and derive the transition probability for arbitrary types of recombination events. Using this, we calculate the run-length distribution of 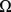 along recombining chromosomes. Finally, in Section 0.4, we calculate the average proportion of hidden recombination events and derive its limiting behavior for large sample sizes.

We remind the reader that the spatial coalescent is a non-Markovian process and not completely determined by transitions of any finite order. However, it is a homogeneous process. Therefore, first-order transition probabilities are well-defined and independent of the position in the sequence. Here, we compute first order probabilities for single recombination events from one tree to the next, averaging over all trees of the ARG which are not directly involved in the recombination event considered. Therefore, our results hold for the spatial coalescent as described by the ARG [13]. In fact, the ARG is the model which is underlying all our calculations.

## Results

### (a) Tree Distribution and Recombination

We consider a sample of 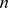 “chromosomes” from a diploid panmictic population of constant size 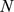. Without recombination, the genealogical history for these chromosomes is described by the classical coalescent process [1], [2]. The set of all possible coalescent trees of size 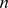 is a product 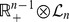, where 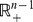 contains positive real waiting times of 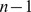 independent coalescent events and the discrete set 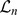 represents the set of all possible tree topologies. For our purposes here it is more convenient to consider labelled coalescent trees: this means that not only the internal nodes are ordered but also the leafs carry leaf labels. Hence [19] (see also http://oeis.org/A006472), the cardinality of 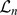 is
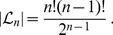
(1)


Furthermore, all trees in 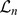 have the same probability 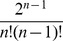, when they are generated under the standard coalescent process [20]. The waiting times 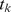 for a coalescent event, given 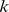 lineages, are exponentially distributed with mean 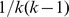. Time runs backward from the leafs to the root of the tree and is measured in units of the coalescent, i.e. time is scaled by four times the population size. Therefore, 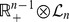 can be regarded as being equipped with a probability mass function which factorizes into a probability density 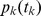 for each waiting time (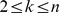) and the discrete probability for the topology 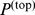. For trees 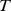 in the above sense, we denote the resulting probability ‘density’ by
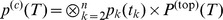
and we have
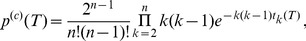
(2)where 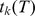 is the time interval during which the coalescent tree 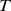 has 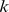 lineages.

Modeling recombination as an ARG [13], there are two processes to be considered: coalescence and recombination. Given 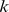 independent lineages, in the coalescent process two lineages merge into a single one with rate 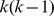. In the recombination process, a single lineage splits into two with rate 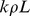, where 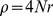 denotes the population recombination rate, 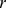 is the recombination rate per base and 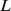 is the finite length of the sequence. After a recombinational split the two ancestral lineages correspond to different sequence fragments, left and right of the point of recombination. This point is chosen uniformly along the sequence of length 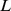. We assume that 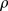 is small, so multiple recombination events in the same position are negligible.

Given a tree 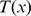 in position 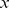, the length before the first recombination event downstream (or upstream) of 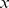 is geometrically distributed with parameter 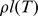, where 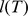 represents the total length of the tree. Since 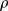 is small, it can be safely approximated by an exponential distribution with the same parameter 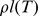.

**Figure 3:**
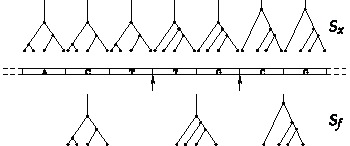
Distinction between sequences 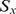 and 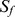 along a recombining chromosome (sketched in the middle). Sequence 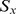 is the sequence of coalescent trees plotted for each nucleotide. Sequence 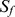 is the sequence of coalescent trees for each recombination fragment. Recombination breakpoints are indicated by arrows.

Recombination events may change the shape of the tree. The local tree at position 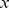 in the genome may differ from the local tree at position 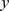 due to recombination. Moving along the genome, we consider two different sequences of trees: the sequence 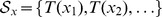 of local trees for all positions 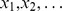, and the sequence 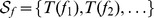 of local trees which are separated by a *single* recombination event (Figure 3). Note that a tree in 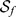 can span several base positions, as the typical length 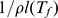 of the fragment 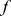 is greater than 1. Also, note that consecutive trees in 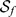 need not be different. This occurs when fragments are separated by hidden recombination events.

The standard coalescent without recombination is recovered when looking at the tree for a single position 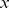 in the sequence, ignoring all other trees. Neither the rate of coalescent events nor the choice of coalescing lineages in this tree are influenced by ancestral lineages at other positions. The local tree 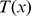 at any position 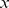 is therefore a standard coalescent tree without recombination [21] and the marginal density of a tree in position 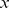 of the ARG is identical to 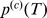; i.e., picking the tree in position 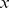 from a random sequence 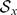 is equivalent to generating one from the standard coalescent process without recombination.

**Figure 4:**
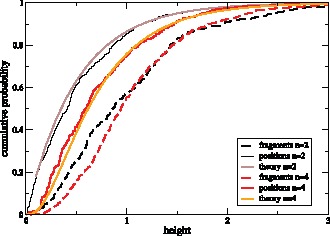
Cumulative distribution of tree height for 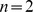 (black) and 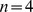 (red) along a recombining chromosome of length 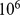 bp. Shown are the height distribution of trees in 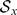 (solid; “positions”) and in 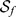 (dashed; “fragments”). For comparison, the theoretical distributions for 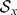 are plotted in light colors.

**Figure 5:**
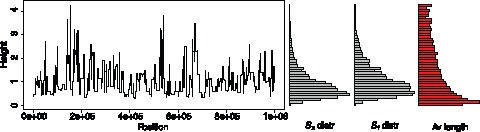
Height of neutral coalescent trees along the genome. One simulation run using *ms* [5] with 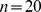 and 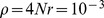. On the right, the distribution of the trees according to 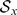 and 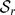 and the average length before a recombination event, for a simulation of a sequence of length 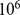.

On the other hand, picking a tree from a random sequence 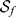 results in a different distribution. The reason is that short trees recombine less, therefore they tend to span larger regions and to be under-represented in 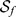 compared to 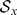, as illustrated in Figures 4 and 5.

In fact, the two distributions differ by weights which are proportional to the length 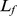 of the fragments spanned by each tree. Since in the limit of large sequences the average length is 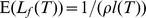, we have 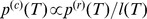. Therefore, for large sequences, the tree density after a random recombination event is given by
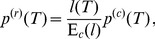
(3)where 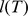 denotes the total length of the tree. For the standard neutral model, 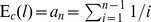. Note that the two distributions differ only in their weights of branch lengths, but not with respect to topology.

The argument leading to eq (3) can be made rigorous under the assumption of infinitely long chromosomes, using the fact that the coalescent with recombination is an ergodic process [22] (see Text S1, Supporting Information eqs (1)–(3)). As a check of eq (3), we show that 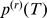 is invariant under a single recombination event. Let 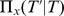 be the transition density from tree 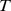 in a given position 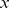 to tree 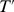 in position 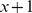, and 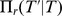 the transition density from tree 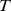 to tree 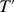 obtained by a single recombination event. Since the marginal density 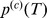 is the same for every position, we have
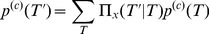
(4)independent of the recombination rate. For small recombination rates and at first order in 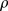, we have 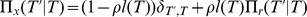. Substituting this into (4) gives



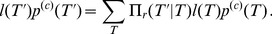
(5)That is, after normalization 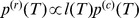 is an invariant distribution under 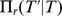. The normalization is 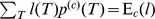.

Furthermore, any marginal tree obtained from an ARG (conditioned on the number of recombinations in the sequence) by choosing randomly an ancestral lineage for every recombination event is distributed according to 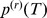. This can be seen from symmetry: none of two trees separated by a single recombination event is distinguished, so they have the same distribution, which is the invariant distribution under a single recombination event, i.e. 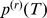. This property has far-reaching consequences since it makes it possible to exploit the symmetries of the ARG.

Note that the two distributions, 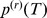 and 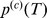, become asymptotically identical when 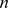 becomes large. To see this, it suffices to consider the random variable 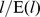. Its mean is identical to 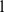. Since 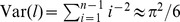 for large 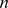 [2], one has
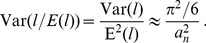
(6)


The right hand side of equation (6) converges to 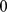 with increasing 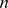. Therefore the factor 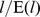 converges to 1 and 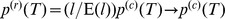 (in the sense of local weak convergence). The relations between the empirical probability distributions 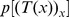 and 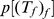 along the sequence and the probability densities 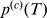 and 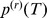 are summarized in the following diagram:
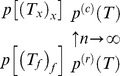



The distributions 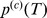 and 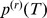 need to be carefully distinguished when measuring the effect of a single recombination event. If one asks for the first recombination event downstream of a given position 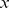 in the genome, then the initial tree at position 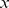 is distributed with 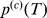. If one asks instead for the effect of a randomly chosen recombination event, then the density 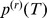 is the appropriate one.

### (b) Height-changing Recombination Events

#### Probabilities of height changing events

Recombination can be interpreted as a random prune-and-regraft event on the tree [23]. First, a time point of pruning is selected uniformly anywhere on the tree; second, the node immediately above the selected branch is removed; third, the pruned branch is re-grafted onto the tree anywhere above the pruning point or onto the ancestral lineage of the root, forming a new node. For hidden recombination events, prune and re-graft occur on the same branch, without modifying topology or branch lengths of the tree.

**Figure 6:**
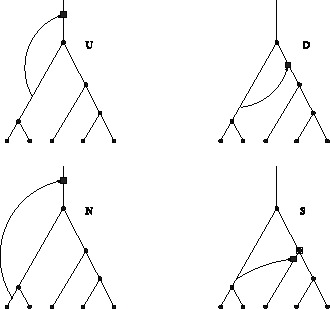
Types of height-changing recombination events. The square indicates the new node created by re-grafting. It forms the new root in cases U, D and N. In case S, an existing internal node becomes the new root (empty square overlaid on node 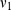).

We denote the root node by 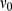 and the first internal node by 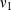. There are four types of recombination events that change the height of the tree (Figure 6).

U (‘up’): a prune-and-regraft event on the root branches generates a higher root without changing the topology;

D (‘down’): a prune-and-regraft event on the root branches generates a lower root without changing the topology;

N (‘new’): pruning a branch below the root branches and re-grafting onto the ancestral branch of the root creates a new root, while the old root becomes internal node 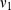;

S (‘substitute’): pruning a root branch and re-grafting onto a branch in the subtree of 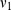 causes 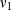 to become the root.

In fact, for the root to change height it must either be shifted (cases U and D) or be replaced (cases N and S). If the root is replaced, it can become an internal node 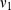 (case N) or be lost (case S). Cases U and D leave the topology unchanged, while cases N and S do not.

We denote the probabilities of these events by 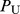, 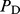, 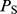, 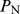. We compute these quantities under both distributions, 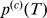 and 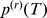.

**Figure 7:**
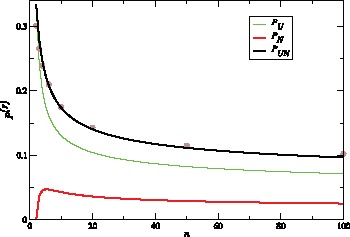
Increase of tree height. Probabilities 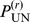 (black), 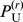 (green) and 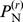 (red) of events that increase tree height as a function of sample size 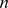. Dots represent the values of 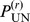 obtained by simulations using program ms [5] and selecting a random recombination event which is far from the sequence boundaries.

Given a coalescent tree of size 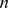, let the *level* 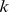 be the time interval when exactly 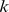 independent lineages coexist, with 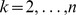. The waiting time at the 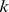
th level is 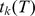, in the following called 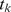 for short. Tree height may be increased by recombination events of type U or N. The total probability for this, 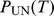, is given by the sum of the probabilities of pruning at all possible levels, but never re-grafting lower than the root:
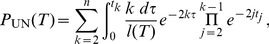
(7)where the product is defined to be 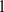 when 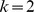. This is a telescopic series that can be re-summed in a function of the total length of the tree



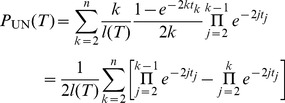
yielding the simple result 
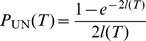
(8)Interestingly, this probability depends only on the total length 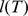 of the tree and not on the topology. Very short trees grow with high probability, very long trees are unlikely to grow (Figure S1). The average probability of height-increase when passing from one recombination-delimited sequence fragment to the next is
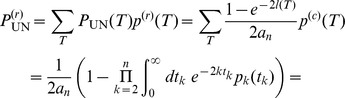


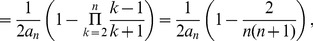
(9)which agrees very well with simulations (Figure 7). Note that 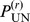 approaches zero as slowly as 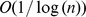.

This result can also be derived directly by counting ARGs, since 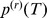 corresponds to the distribution of a random tree in an ARG. We will consider the case of a recombination event at a given level 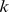 and then average over all levels. To obtain the total number of ARGs 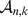 with a single recombination event at level 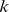, choose a tree at random (among 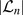 possibilities), then choose the branch to be pruned (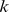 possibilities) and the branch to which it is re-grafted at the same or a higher level (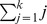 possibilities). Therefore,
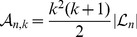
(10)The number of ARGs where the new tree is higher than the old one is 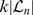, because there is just one possibility of re-grafting, namely on the ancestral lineage above the root of the old tree. The probability of pruning at level 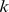 in the old tree is 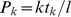. Therefore, one can average over 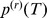 to obtain 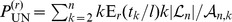, which is identical to equation (9).

Focusing now on pruning of the root branches, we obtain 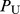 analogously to equation (7). Let 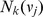 be the number of direct descendants of node 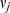 at level 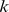. 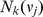 can take values 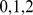. The average value of 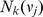 satisfies the recursion
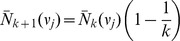


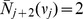
that has the solution



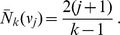



In particular, the average number of direct descendants of the root at level 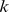 is 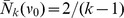. The probability 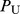 is a modification of equation (7): multiplying by the fraction of events that are actually of type U, i.e. 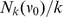, one obtains
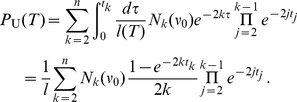
(11)


In contrast to equation (7), equation (11) cannot be easily simplified since it depends also on the topology. After averaging over 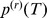, we obtain
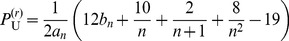
(12)and
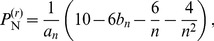
(13)where 
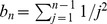
.

The probabilities 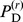 and 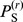 can be computed similarly to the above formulae, giving
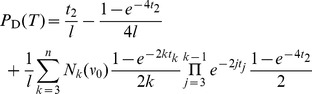
(14)and



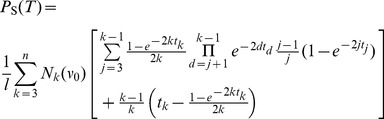
(15)(Text S1, Supporting Information eqs (4)–(9)). Alternatively, one may employ an argument based on symmetry properties of the ARG. Among two adjacent trees in the ARG, the left one is smaller or larger than the right one with equal probability. Therefore,
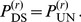
(16)


The same is true when the root is only shifted. Thus,
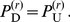
(17)


Hence, by subtraction,
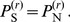
(18)


Note that the identities (17) and (18), being topological in nature, are also valid for models with variable population size. A related result about the probability that a random recombination event leaves tree height unchanged (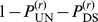) has been obtained previously by Griffiths & Marjoram [24].

Equations (8), (11), (14), (15) are valid also when averaging over the distribution 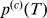, instead of 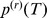. However, exact results are available only for small sample sizes. For the case of arbitrary 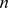 we use the following Taylor approximation of the ratio moment
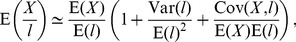
(19)where 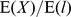 represents the desired probability 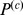. When the expansion is truncated at zeroth order (i.e., replacing the first moment of the ratio by the ratio of first moments), one obtains the results analogous to equations (12), (13), (17) and (18). More detailed calculations are given in Text S1, Supporting Information eqs (10)–(12). These yield, for instance, the probability of increasing tree height



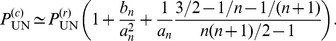
(20)Note that the scaling factor on the right hand side in equation (20) approaches 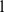 very slowly with increasing 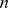. The case 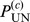 is actually an exception since an exact formula exists [15] for all values of ; in fact, 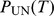 depends only on 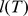, therefore it is sufficient to average this quantity over the distribution of 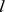 obtained in [15]. For small samples there is a considerable difference between 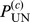 and 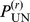. For example, if 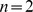, we have 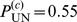 while only 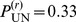.

#### Amount of change in height

The variation in height 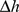 has a simple distribution. If the height increases, then the difference is given by the waiting time for coalescence of two lineages. It is
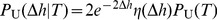
(21)and
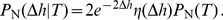
(22)where 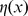 is the Heaviside function, 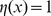 if 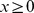 and 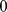 otherwise. If the height decreases because of an event of type D, its distribution is given by the waiting time for coalescence before time 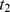, equivalent to the “bounded coalescent” for two lineages [25]



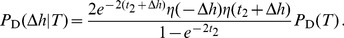
(23)For events of type S, the variation in height is simply the waiting time 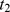 of the tree
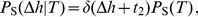
(24)where 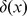 is the Dirac delta distribution. Averaging these quantities over 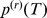 and using the symmetries of the ARG, we obtain
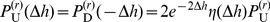
(25)and
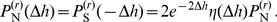
(26)i.e., all these variations in height are exponentially distributed for an average tree.

Taking expectations, the average change in height after one of these events is
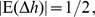
irrespective of the type of event, i.e 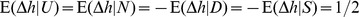. Comparing this to the average height of a tree, 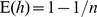, one notices that a single recombination event changes tree height by 50% on average.

### (c) Root Imbalance and Recombination

Let 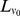 (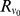) be the number of left (right) descendants of the root. We have 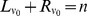. We call the random variable 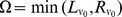 *root imbalance*. 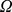 is a coarse-grained measure of tree topology. A recombination event may or may not change 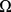 and a change of 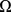 is neither sufficient nor necessary for a change in tree height. Since many recombination events induce rearrangements of the lower branches (close to the leafs) of the tree, they may affect 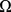 without affecting tree height. Still, large changes in 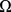 are often associated with height-changing recombination events of type N or S and thus are associated with drastic changes of tree topology.

In this section we calculate the transition probabilities 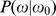 for 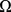 under a single recombination event, averaged over the initial tree. First, we focus on events of type UN, i.e. increasing height, and then we obtain the transition probabilities for all types of events separately.

#### Root imbalance and height-increasing events

Let the *size* of a branch be the number of leaves below the branch. A specific tree of size 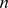 can be fully described by the probability 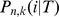 that a randomly chosen branch at level 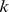 has size 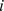. Averaging over trees of size 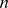, the probability that a branch of level 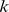 has size 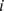 is
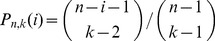
(27)


[26]. Let 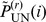 be the probability that the height increases and the pruned branch has size 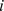. It is obtained, similarly to 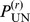, by multiplying each term of the sum in equation (7) by 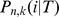. Thus, given a tree 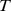,
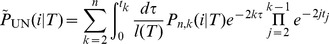
(28)and, averaging over 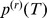, one obtains
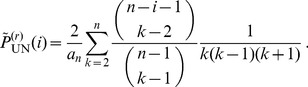
(29)More generally, the probability that the pruned branch has size 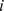, given that recombination leads to an increase in height, is simply 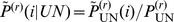. The random variable 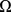 can take values between 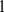 and 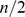 and is the folded version of the random variable 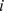 which ranges from 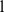 to 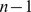. Hence, the distribution of 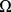, after an event that increases tree height, is



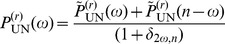
and the distribution of 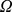, conditioned on tree height increase, is
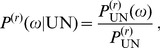
(30)as illustrated in Figure S3.

Now we calculate the probability conditioned on the value 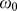 of 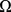 before recombination, i.e. the transition probability 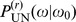. The basic quantity for this computation is the probability 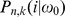 that a branch at level 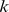 has size 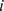 in a tree of total size 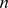, given that the size of the root branches are 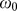 and 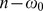. To compute this, we need information about the actual size 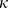 at level 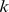 of the subtree of size 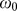 of the root. We denote the distribution of 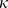 by 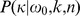 and the distribution of 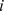 given the sizes 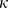 and 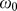 of its root subtree at levels 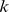 and 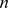 by 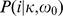. Note that 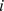 does not depend on 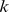 nor on 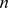, but only on the size of the root subtree to which it belongs (see Figure S4). Therefore we have
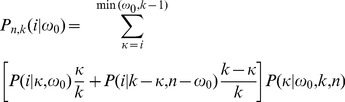
(31)The probability 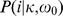 is equal to
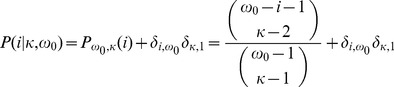
(32)as can be shown by considering the corresponding subtree of the root as the whole tree and using equation (27). The probability 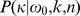 depends only on the topology, therefore it can be obtained by counting the number of labelled coalescent trees (http://arxiv.org/abs/1112.1295v2) with a root branch of size 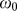 in the whole tree that reduces to size 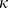 at level 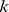, denoted by 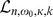, and dividing by the total number of trees with a root branch of size 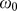, denoted by 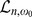. Using that 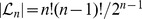, that the coalescent process induces a uniform distribution on 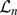 and that the distribution of 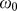 is 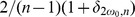 [27], we have



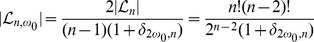
(33)The set of all trees in 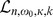 can be generated in the following way: (i) choose 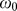 leafs out of 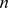; (ii) choose an relative order of the 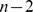 coalescent events among the two subsets with 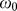 and 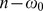 leafs such that among the first 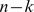 events 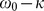 events belong to the first subset and 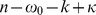 belong to the second; (iii) choose a topology for the root subtree of size 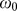; (iv) choose a topology for the complementary subtree of the root. This process generates exactly once all trees in 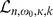, except for the case 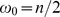, where each tree is generated twice. Therefore, we have
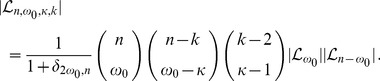
(34)


Taking the ratio of tree counts, we obtain an hypergeometric distribution
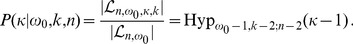
(35)


Finally, inserting the results (32) and (35) into (31), we obtain
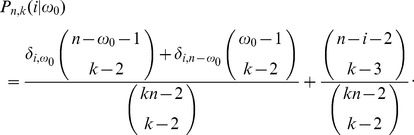
(36)

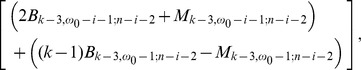
where 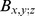 and 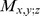 are the normalization and the mean (i.e., the zeroth and first moment) of the hypergeometric distribution with parameters 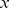, 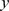 and 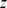, if they satisfy 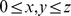, and 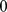 otherwise. Note that 
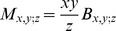
.

As before, we introduce 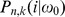 in equation (7) to obtain
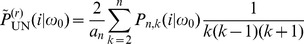
(37)and, finally, the result



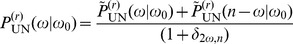
(38)

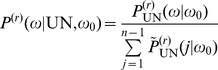
(39)


**Figure 8:**
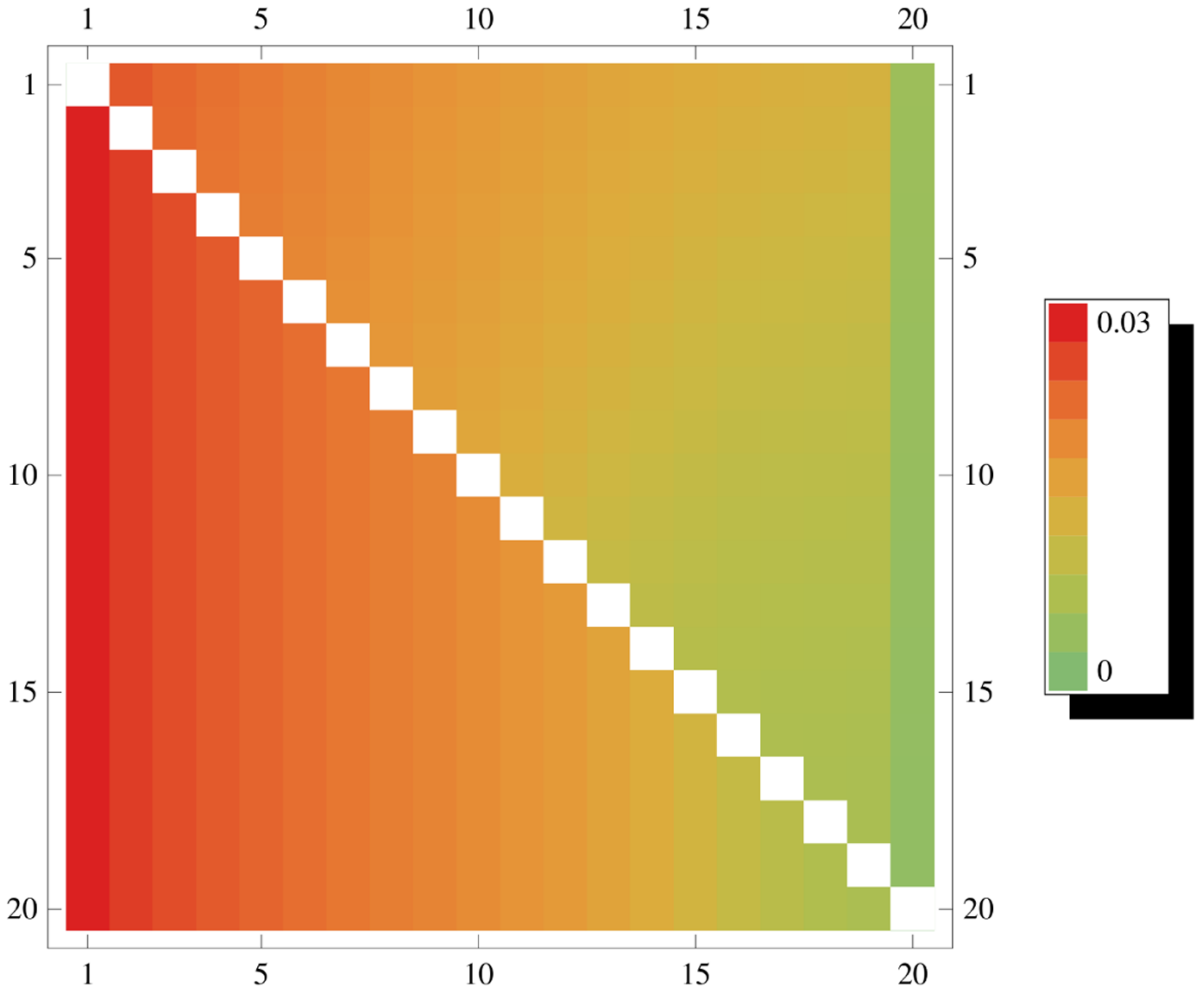
Transition probabilities of 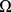. Distribution 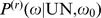 as a function of 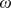 (horizontal axis) and 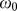 (vertical axis) for 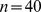. The diagonal terms (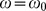) are not shown.

Figures 8 and S5 illustrate these probabilities. With a recombination event of type N, 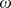 tends to change to smaller values. Thus, the tree becomes more unbalanced. However, by far the highest probability is attained for 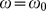, irrespective of 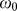 and mainly due to events of type U. This case is omitted from the figures for clarity.

#### Other recombination events that change root imbalance

Now we consider all possible recombination events that change 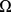. Events of type U and D do not change 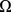, so they can be ignored. Apart from the events of type N that we discussed above, other relevant recombination events are of type S and of type R (‘root remains’), i.e. any event which leaves the root untouched. To compute the probability of a change in 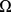 for these types of events, we use the fact that random trees from an ARG have the distribution 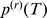 and that the probability of each labelled ARG topology is the same. Due to this, we need only count the number of ARGs with a single recombination event at level 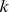 compatible with root imbalances 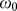 and 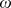, and denoted by 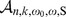 and 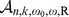. Then, we divide by the total number 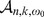 of ARGs with a recombination at level 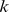 and root imbalance 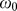 for the original tree. Putting everything together, we obtain
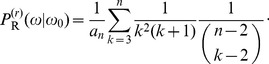
(40)

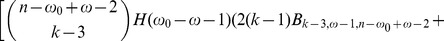


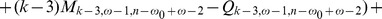


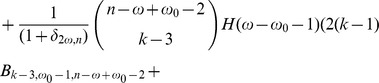


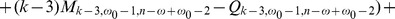


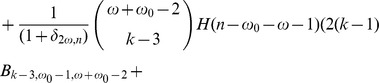


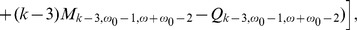
where 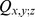 is the second moment of the hypergeometric distribution with parameters 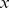, 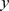 and 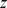 satisfying 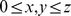, and 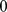 otherwise, and 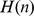 is the Heaviside function, 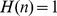 if 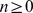 and 0 otherwise. Note that the ARG symmetries imply the non-trivial relation



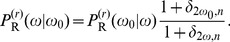
(41)The relative importance of 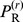 versus 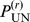 and 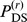 is shown in Figure S6.

The contribution for events of type S can be obtained using the symmetry properties of the ARG. In fact, an ARG with a recombination event of type S changing 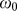 to 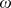 is equivalent to an ARG with an event of type N changing 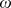 to 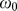. Therefore,
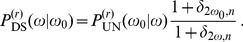
(42)


This result is essentially the transpose of the one shown in Figure 8, i.e. after an event of Type S, 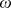 has an almost uniform distribution irrespective of 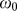.

Finally, the transition probability is
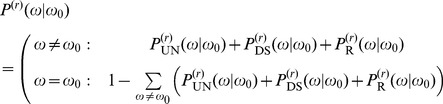
(43)


This distribution is shown in Figures S7 and S8 for 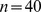.

### (d) Hidden and Silent Recombination Events

Counting ARGs we now determine the fraction of *hidden* recombination events, i.e. those which neither change tree topology nor branch lengths. Since these events are ‘invisible’ when analysing sequence polymorphisms or haplotype structure, their frequency can only be estimated by theoretical means.

Hidden recombination events are caused by pruning and re-grafting on the same branch (see Figure 2D). Let 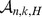 denote the number of ARGs with a hidden event at level 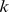. Since ARG topologies are equiprobable under 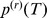, the probability that a recombination event is hidden is
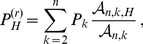
(44)where 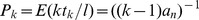 is the probability of pruning at level 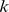. To calculate 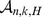 we need to consider the following ingredients. A branch pruned under node 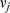 can be regrafted in 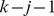 topologically inequivalent ways on the same branch (but possibly on different levels). This number has to be multiplied by the number of branches under node 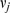 at level 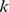 (denoted by 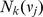). Then, one has to sum over all possible nodes 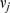 and over all possible initial trees 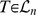. This yields



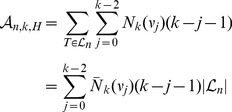
(45)Combining eqs (44) and (45) we obtain
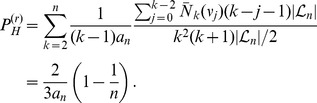
(46)


This means that the fraction of hidden recombination events is of the order 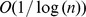. They are quite frequent for small to moderate 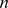, but become increasingly rare with increasing 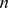. Still, even when 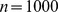, about 9% of all recombination events are hidden.

Using the same technique of counting ARGs also the fraction of silent recombination events (i.e. events that do not change topology but that may change branch lengths) can be obtained. We start by counting events that are silent but not hidden. Given a tree, select a branch for pruning. Then, there are exactly two ways for re-grafting: either on the branch immediately above or on the branch immediately below the old parent node of the pruned branch (Figure 2B or C), but not on the pruned branch itself (the latter would be a hidden event). Performing similar calculations as before we obtain
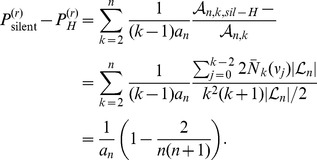
(47)


Therefore,
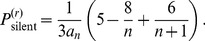
(48)


Note that the following holds:
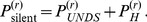
(49)


An intuitive explanation is the following: for any pruning point, there are two possible ways for re-grafting such that tree topology remains unchanged and there is exactly one way for re-grafting which leads to an increase of tree height. Therefore, 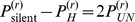. Then, eq (49) follows from symmetry of the ARG. Note that this argument is topological and does not depend on waiting times, i.e. branch lengths.

### (e) Correlation Lengths

**Figure 9:**
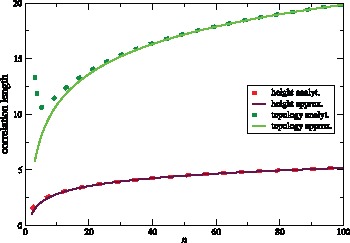
Correlation length 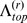 (blue line) as a function of sample size 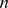. The red line is the approximation 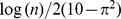.

Since the spatial coalescent is a non-Markovian process, it is important to know over which chromosomal distances correlation and statistical dependence among trees persist. Correlation between trees, measured by any well-behaved tree statistic, decreases with distance. An interesting question is how quickly recombination reduces correlation. The answer depends on the particular statistic which is employed to measure correlation. Topology based statistics, such as 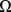 (measuring imbalance at the root) or Colless’ index [28] (measuring imbalance at all internal nodes), behave differently from length based statistics, such as tree height (Figure 9).

We use our above results regarding events of type U, D, N, S and R to give a quantitative answer. The idea is to approximate the correlation length for a statistic by the inverse of the probability of recombination events that have a strong impact on this statistic.

Events of type U or D change height, but leave the topology unchanged. Events of type R preserve height but alter topology. Events of type N or S may change both, height and topology. They also lead to the fastest decay of correlation.

The average number of recombination events before an event of type N or S occurs is the inverse of this probability. This quantity is a rough estimate for the correlation length of tree shape. The numerical values of 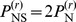 for 20≲*n*≲100 lie between 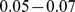 (Figure S2). Based on this estimate, correlation between trees should decay strongly within 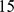 to 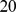 recombination events. This is in agreement with numerical simulations. More generally, the topological correlation length can be roughly estimated as
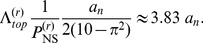
(50)It Increases Logarithmically in 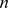 (Figure 9)

To translate this into physical length, we assume that the distance between two consecutive recombination events is exponentially distributed with mean 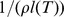. Averaging over 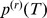 we obtain 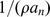. Therefore, distance 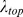 between two events of type N or S is approximately
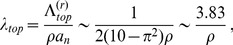
(51)independent of 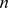. For example, if the scaled recombination rate is 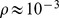, the genomic distance between such events is about 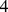
kb. Assuming that also the scaled mutation rate is 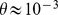 per bp and assuming 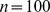, an interval between drastic recombination events of type N or S contains about 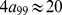 polymorphic sites. This number should be sufficiently high to enable at least a rough tree re-construction from SNP data, and to estimate 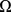. It will probably not be sufficient for the reconstruction of the fine topological structure of the lower branches.

To estimate the correlation length of 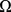, also events of type R need to be taken into account. In fact, changes in 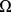 occur more often than events of type N or S. Using equation (43), we determined the run-length of 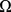, i.e. the number of recombination events that occur before a change in 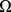 happens. Considering a random initial tree, an estimate for the run-length is given by
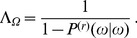
(52)


**Figure 10:**
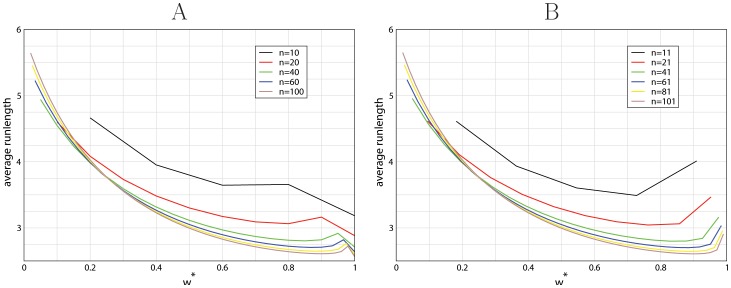
Run length 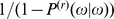 as a function of 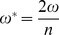 for even sample sizes (A) (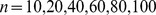) and for odd sample sizes (B) (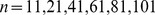).

The run-length is longer for more imbalanced trees, but always on the order of a few recombination events (between 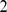 and 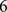; Figure 10). This is also a reasonable estimate for the correlation length of the fine topological structure.

We now consider correlation in tree height. Height can change by events U,D,N and S. The average change in height is the same, 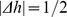, for all these events. Therefore, correlation length can be estimated as
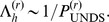



Since


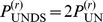
 is between 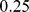 and 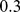 for 20≲*n*≲100 (Figure 7), drastic changes in height are expected on average every 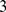 to 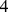 recombination events. More generally, the correlation length also increases logarithmically in 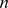 and is
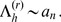
(53)


For the physical correlation length we have.
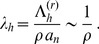
(54)This is only about a quarter of the topological correlation length. Therefore, an exact reconstruction of tree height is difficult. For instance, for 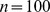 and 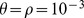, one would have on average only 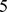 SNPs to estimate height or other tree parameters.

For the case 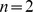, Hudson [21] gives a formula for the correlation between the heights of two trees in dependence of the recombination rate 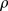. The formula predicts that the correlation drops to about 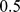 with 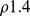, i.e. after approximately 1.4 recombination events. Our rough estimate for the correlation length in this case is 
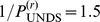
, and in good agreement with Hudson’s result.

Finally, we briefly comment that linkage disequilibrium and haplotype block size depend strongly on the number and distribution of mutation and recombination events along coalescent trees, i.e. they depend strongly on tree topology and length. Since topology can in practice only be indirectly estimated from polymorphism patterns, not all changes in topology are actually visible for these statistics. The correlation lengths estimated from experimental data will tend to be larger than the theoretical estimates presented here. Assuming that haplotype blocks are mostly delimited by ‘drastic’ recombination events, involving a change of topology, we estimate the size of these haplotype fragments 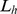, centered at some position 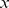 with a tree 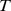. Assuming further that neither tree length 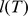 nor the probability of topology-changing drastic recombination events 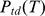 change much after a ‘non-drastic’ recombination event, the probability distribution for the haplotype sizes is
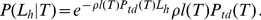
(55)


The average size is then
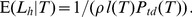
(56)


The class of drastic recombination events that should be considered to determine 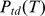 is probably larger than the class of type N and S events. However, 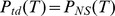 is a reasonable lower bound approximation.

## Discussion

We have considered the effect of single recombination events on coalescent tree topology and explicitly determined the probability with which recombination triggers ‘drastic’ changes. We consider a change to be drastic if it leads to a change of tree height or of tree imbalance. These types of events are of practical interest because both have an effect on the pattern of polymorphic sites which are informative for genealogical reconstruction and evolutionary inferences. The primary effect of height change is upon the number of mutations, while a change in tree imbalance primarily affects the mutation site frequency spectrum.

Our results show important qualitative differences for the two types. The average change in height is quite drastic per se (50% of average tree height), while the average change in imbalance is quite mild, with large jumps occuring only very rarely. Our results hold for the standard neutral model, i.e. a model with constant population size and without substructure. As such, our results may serve as the analytical reference case for constructing formal tests of the neutral evolution hypothesis. For instance, the probabilities of height or topology change are markedly altered in the presence of selective sweeps, i.e. the fast fixation of a mutant allele due to positive selection. Recombination close to the sweep site, where tree height is severely reduced [29], tends to lead to both a drastic increase of tree height and highly imbalanced trees [16], [18]. In contrast, variable population size leaves a different signature on the probabilities of drastic recombination events. Non-constancy of 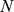 is reflected in branch length variation, but it has no impact on the branching pattern, i.e. on topology. In fact, if panmixis continues to hold, the probability distribution of tree topologies does not depend on population size. Variation of 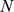 affects only branch lengths and waiting times. Since all our results, averaged over 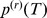, depend implicitly on the first moments of the waiting times through the quantity 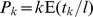, they can in principle be adapted to models with variable population size using the theory developed earlier [26], [30]. A detailed treatment is left to further investigation. Here we just note that the relations (17), (18) and (49) are valid for all models of variable population size.

Population substructure is another important case of deviation from the standard neutral model. Restricted gene flow between sub-populations strongly affects the transition probabilities of root imbalance, but less the distribution of height change. A more detailed discussion of the impact of these evolutionary scenarios upon a test statistic of the neutral evolution hypothesis is given in [18].

We have derived a number of further results which shed more light on the details and consequences of recombination. We analysed the correlation length between trees on a recombining chromosome and showed that topological correlation is generally longer-ranging than correlation in tree height. Still, for both types very few recombination events – on the order of ten – are sufficient to unlink the genealogical histories of two genomic fragments, given standard neutral conditions. The calculations also make clear that correlation length (number of recombinations) scales logarithmically in 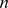. This is important to take into account for deep sequencing association studies.

It is perhaps surprising to see that a considerable fraction of recombination events remains hidden. Even for large sample sizes, about 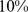 of the recombination events are not visible. An even larger fraction is silent, i.e. does not cause topological changes of the underlying genealogy.

Analyzing root imbalance in more detail, we found that the distribution of 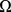-run lengths is biased towards unbalanced trees: under the standard neutral model, unbalanced trees tend to span larger genomic regions than balanced trees. Interestingly, the 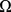-run length, when normalized, is asymptotically independent of 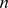. Our results provide a basis to tackle problems of correlation between tree statistics in coalescent models. They extend known results, such as the one by Hudson [21] concerning tree height correlation, to the more general case of arbitrary sample size 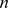.

Some of the quantities studied here involve counting problems of ancestral recombination graphs with a single recombination event. These problems are related to counting problems of phylogenetic networks [31]. Unlike counting problems of trees, which can often be tackled by generating function techniques ([20], arxiv.org/abs/1112.1295v2, arxiv.org/abs/1202.5668v3), only few results are available for tree-like structures with independent cycles so far [32]. Our results represent a step towards a combinatorial treatment of these problems.

## Supporting Information

Figure S1
**Probability of increasing height after a recombination event as a function of the total tree length 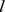.**
(PDF)

Figure S2
**Probability of recombination events 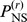 which change tree height and topology as a function of the sample size 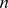.**
(PDF)

Figure S3
**Distribution 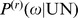 of 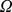 after an event that increases tree height, for 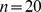.**
(PDF)

Figure S4
**Illustration of the sizes 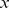 and 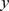 of the subtrees at the levels 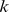 and 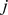 corresponding to pruning and regrafting, respectively.**
(PDF)

Figure S5
**Probability distribution 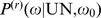 for 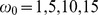 (in blue, pink, yellow, green) and 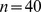. For clarity, only the probabilities for 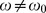 are shown.**
(PDF)

Figure S6
**Ratio 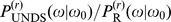 as a function of 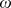 (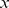-axis) and 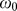 (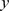-axis) for 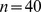.** For clarity, only the probabilities for 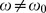 are shown.(PDF)

Figure S7
**Distribution 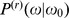 of 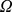 for 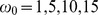 (in blue, pink, yellow, green) and 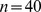.**
(PDF)

Figure S8
**Distribution 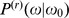 as a function of 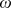 (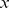-axis) and 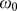 (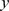-axis) for 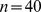. For clarity, only the probabilities for 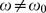 are shown.**
(PDF)

Text S1 **Supporting information.**
(PDF)
