# Supplementary figures and images for: The Effect of Single Recombination Events on Coalescent Tree Height and Shape
Source: PLoS One. 2013 Apr 8;8(4):e60123. doi: 10.1371/journal.pone.0060123 (PMC3620475; doi:10.1371/journal.pone.0060123)

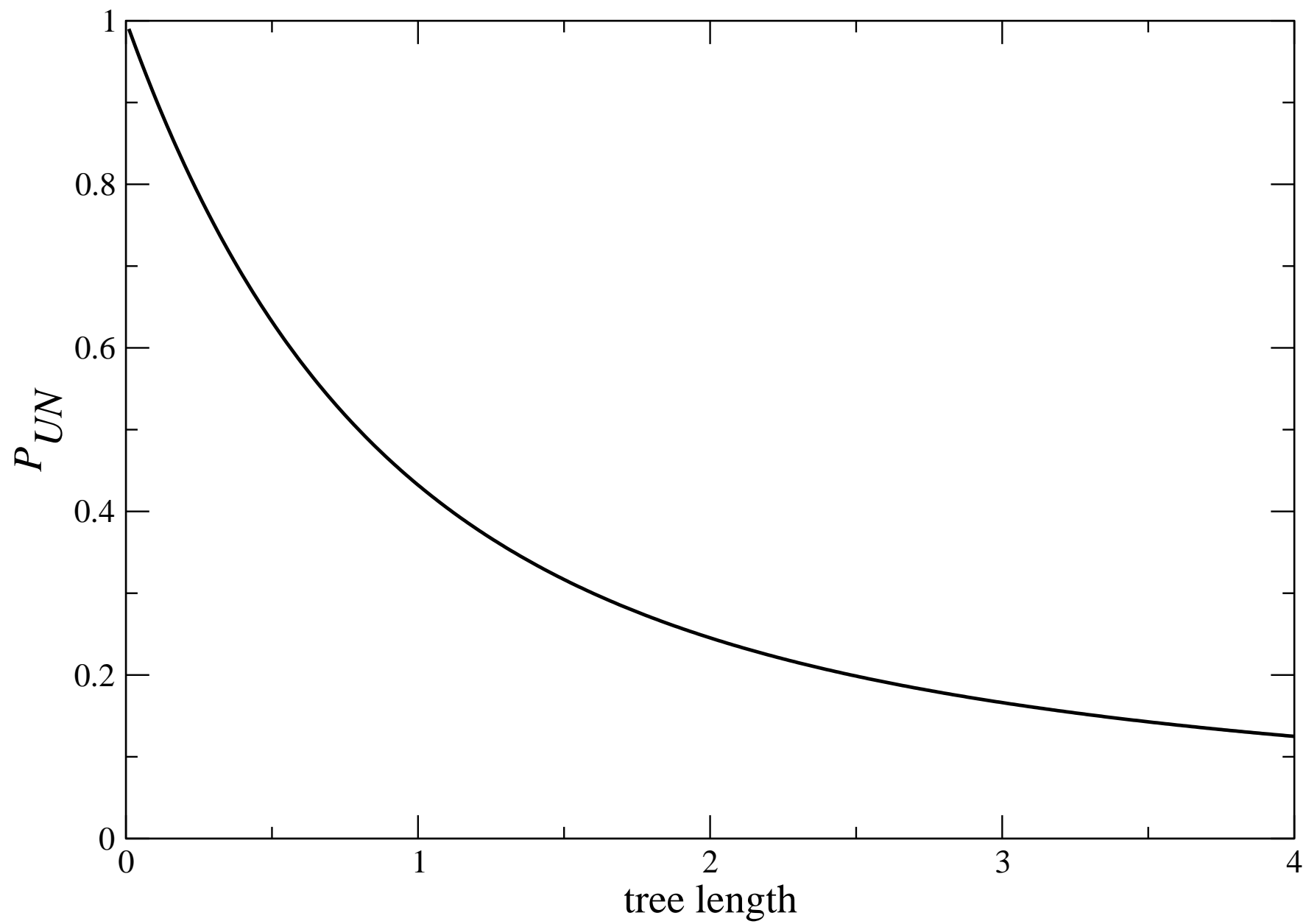

Supplement: Figure S1 — Probability of increasing height after a recombination event as a function of the total tree length . (PDF) [file pone.0060123.s001.pdf]

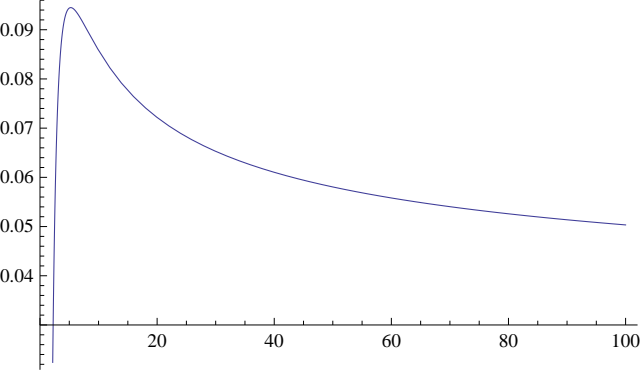

Supplement: Figure S2 — Probability of recombination events which change tree height and topology as a function of the sample size . (PDF) [file pone.0060123.s002.pdf]

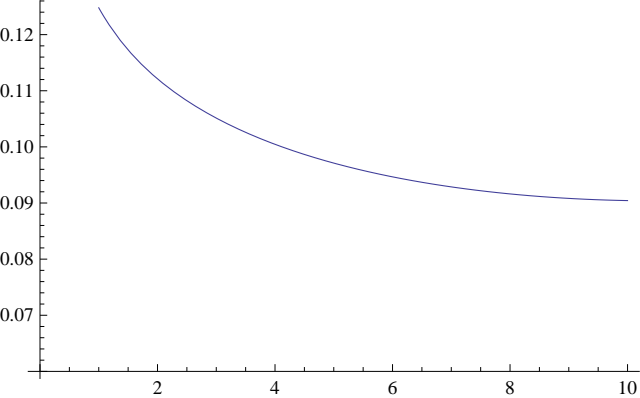

Supplement: Figure S3 — Distribution of after an event that increases tree height, for . (PDF) [file pone.0060123.s003.pdf]

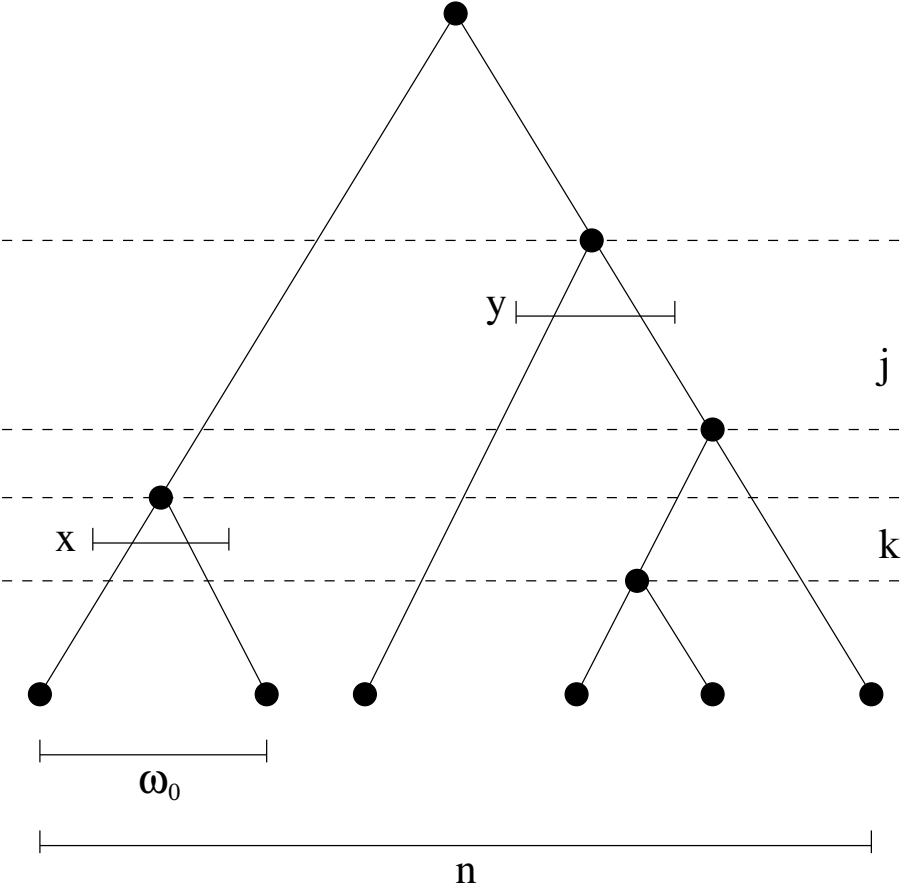

Supplement: Figure S4 — Illustration of the sizes and of the subtrees at the levels and corresponding to pruning and regrafting, respectively. (PDF) [file pone.0060123.s004.pdf]

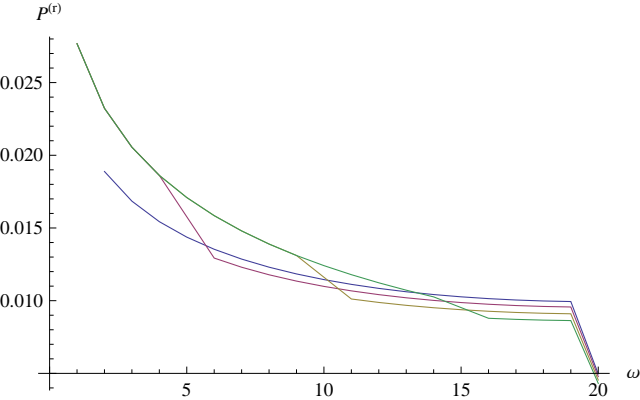

Supplement: Figure S5 — Probability distribution for (in blue, pink, yellow, green) and . For clarity, only the probabilities for are shown. (PDF) [file pone.0060123.s005.pdf]

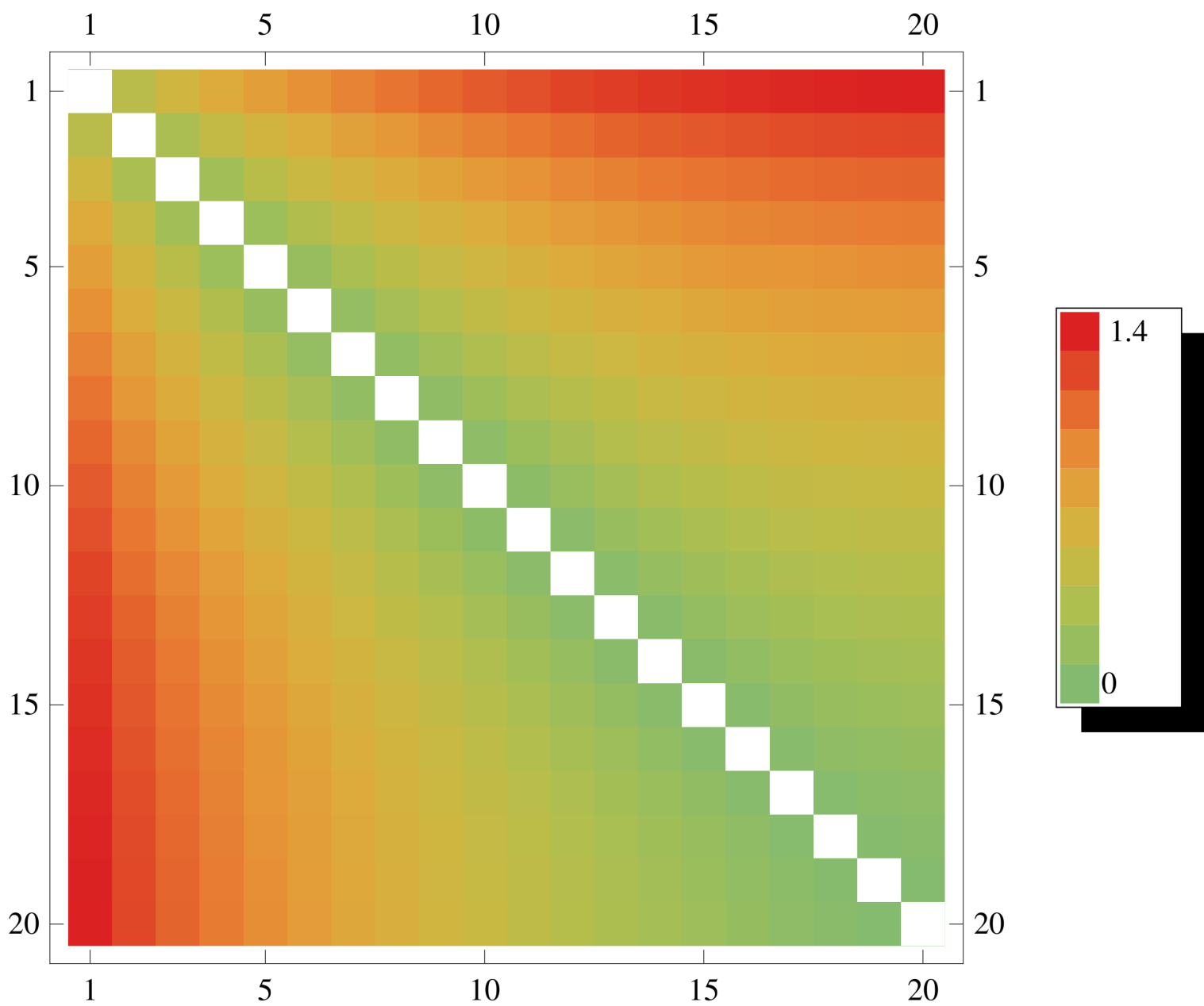

Supplement: Figure S6 — Ratio as a function of (-axis) and (-axis) for . For clarity, only the probabilities for are shown. (PDF) [file pone.0060123.s006.pdf]

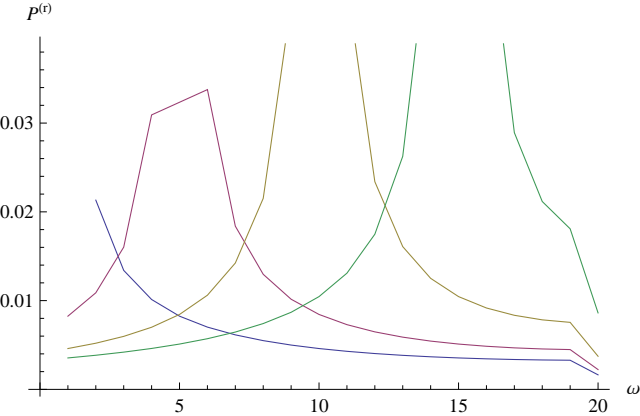

Supplement: Figure S7 — Distribution of for (in blue, pink, yellow, green) and . (PDF) [file pone.0060123.s007.pdf]

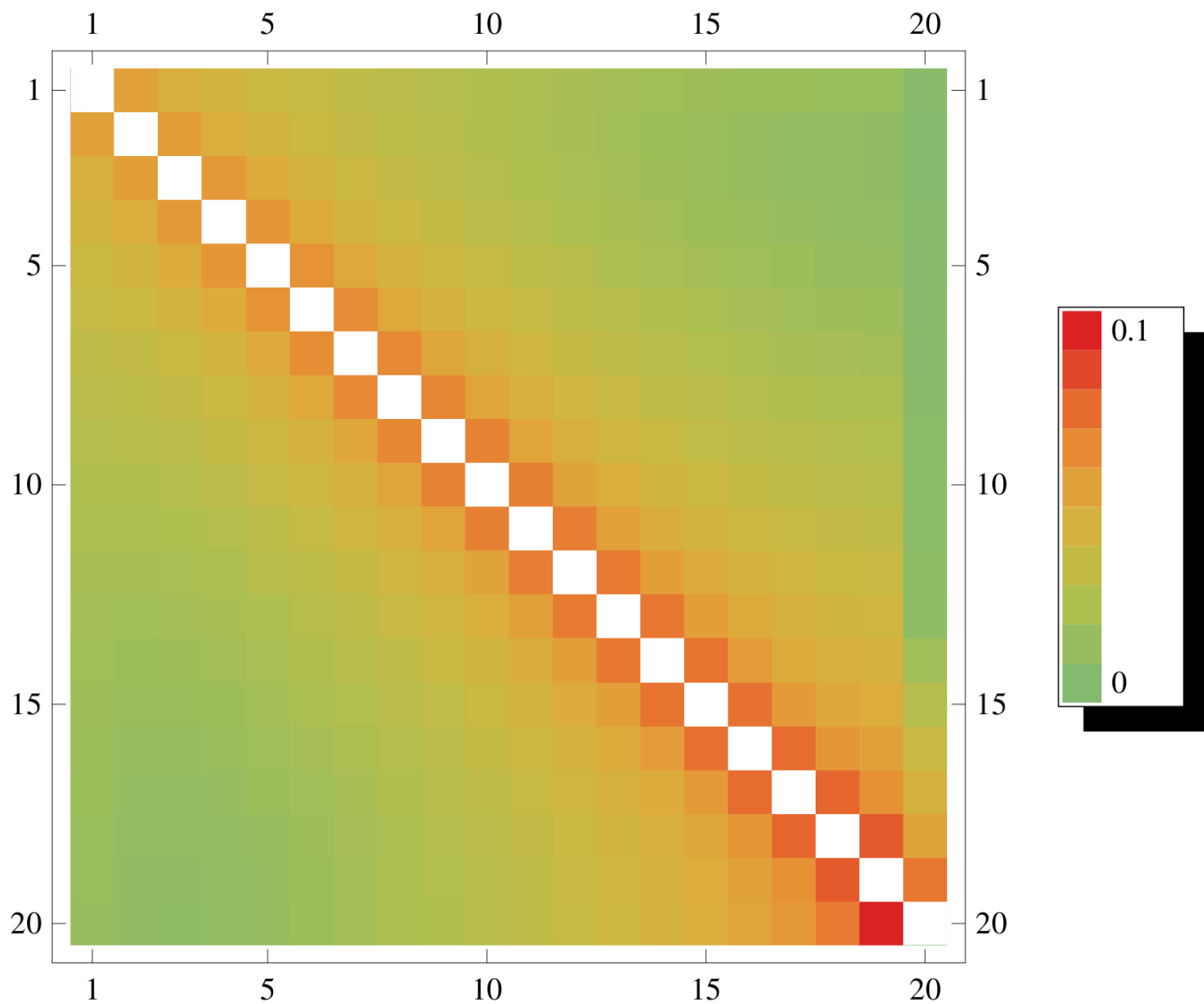

Supplement: Figure S8 — Distribution as a function of (-axis) and (-axis) for . For clarity, only the probabilities for are shown. (PDF) [file pone.0060123.s008.pdf]
