# Supplementary material for: The Effect of Single Recombination Events on Coalescent Tree Height and Shape
Source: PLoS One. 2013 Apr 8;8(4):e60123. doi: 10.1371/journal.pone.0060123 (PMC3620475; doi:10.1371/journal.pone.0060123)
Supplement: Text S1 — Supporting information. (PDF) [file pone.0060123.s009.pdf]

Supporting Information

Sketch of proof of equation (3)

Informally, trees are approximately independent when separated by sufficiently large physical distance on the chromosome or by sufficiently many recombination events. In fact, "knowledge of the history", i.e. correlation between trees, vanishes rather quickly and with only a few recombination events (see Results (e)) and the process is ergodic [22]. Therefore, the empirical distributions of trees along a given sequence S_x (S_f) converge to the probability densities $p^{(c)}(T)$ ($p^{(r)}(T)$) with increasing L.

We denote by $\mathcal{T}$ a measurable set of trees of non-zero measure, by $n_x(\mathcal{T})$ and $n_f(\mathcal{T})$ the number of occurrences of trees of $\mathcal{T}$ in $\mathcal{S}_x$ and $\mathcal{S}_f$, respectively, by E_f the average over fragments in $\mathcal{S}_f$ and by N_f the total number of fragments in $\mathcal{S}_f$. We have

$$\mathrm{E}(n_x(\mathcal{T})/L) \underset{L\to\infty}{\longrightarrow} p^{(c)}(\mathcal{T})\,. \tag{S1}$$

Furthermore,

$$\mathrm{E}(n_x(\mathcal{T})/L) = \mathrm{E}(n_f(\mathcal{T})\mathrm{E}_f(L_f(\mathcal{T}))/L) = \mathrm{E}((n_f(\mathcal{T})/N_f)\mathrm{E}_f(L_f(\mathcal{T}))/(L/N_f)) \tag{S2}$$

and

$$\mathrm{E}((n_f(\mathcal{T})/N_f)\mathrm{E}_f(L_f(\mathcal{T}))/(L/N_f)) \underset{L\to\infty}{\longrightarrow} p^{(r)}(\mathcal{T})\mathrm{E}_r(1/l|\mathcal{T})/\mathrm{E}_r(1/l) \tag{S3}$$

where the convergence follows from ergodicity. Therefore $p^{(c)}(\mathcal{T}) = p^{(r)}(\mathcal{T})\mathrm{E}_r(1/l|\mathcal{T})/\mathrm{E}_r(1/l)$. By inverting this relation and taking the Radon-Nikodym derivative (i.e., passing to the probability density) we obtain (3).

Note that for sequences of finite length, the distribution of random trees in $\mathcal{S}_f$ depends on the length of the sequence. For large sequences it converges to $p^{(r)}(T)$ as discussed above, while in general the distribution interpolates between $p^{(r)}(T)$ and $p^{(c)}(T)$ and for very short sequences it resembles closely $p^{(c)}(T)$. However, here we consider only the limit of large sequences.

Proofs of equations (14), (15) and (40)

Equation (14): Events of type D originated from a pruning event from a root branch to the other. The prune and re-graft can occur at level 2; in this case, integrating over all possible events we obtain a contribution

$$\frac{t_2}{l} - \frac{1-e^{-4t_2}}{4l} \tag{S4}$$

that depends only on t_2. If the pruning occurs at another level, then we have to sum the probability of not coalescing before ν_1 over all possible levels with a root branch (i.e. with $N_k(\nu_0) = 1$) and then multiply by the probability $\frac{1-e^{-4t_2}}{2}$ of a coalescence on the other root branch, obtaining a contribution

$$\frac{1}{l}\sum_{k=3}^{n} N_k(\nu_0)\frac{1-e^{-2kt_k}}{2k}\prod_{j=3}^{k-1} e^{-2jt_j}\frac{1-e^{-4t_2}}{2} \tag{S5}$$

that summed to the previous one, gives eq. (14).

Equation (15): Events of type S originated from a pruning event from a root branch (at level higher than 2) that regrafts on the subtree of ν_1. We have to sum over all possible pruning levels k as in the case above. The branch could regraft at the same level, giving a contribution

$$\frac{1}{l}\sum_{k=3}^{n} N_k(\nu_0)\frac{k-1}{k}\left(t_k - \frac{1-e^{-2kt_k}}{2k}\right) \tag{S6}$$

or at an higher level. In this case we have to sum the probability of regrafting at level j over all levels $2 < j < k$:

$$\frac{1}{l}\sum_{k=3}^{n} N_k(\nu_0)\sum_{j=3}^{k-1}\frac{1-e^{-2kt_k}}{2k}\prod_{d=j+1}^{k-1} e^{-2dt_d}\frac{j-1}{j}(1-e^{-2jt_j}) \tag{S7}$$

that summed to the previous one, gives eq. (15).

Equation (40): We need to count the number of ARGs with a single recombination event at level k compatible with root imbalances ω_0 and ω, which are denoted by $\mathcal{A}_{n,k,\omega_0,\omega,\mathrm{S}}$ and $\mathcal{A}_{n,k,\omega_0,\omega,\mathrm{R}}$, and then divide by the total number $\mathcal{A}_{n,k,\omega_0}$ of ARGs with a recombination at level k and root imbalance ω_0 for the original tree. The resulting probabilities should be averaged over the probability P_k of a recombination event at level k, that is $kt_k(T)/l(T)$, where T is the original tree. Averaging over all waiting times in $p^{(r)}(T)$, we obtain $P_k = 1/((k-1)a_n)$.

Since the time of the recombination event does not depend on ω_0, the number of ARGs $\mathcal{A}_{n,k,\omega_0}$ is simply $|\mathcal{L}_{n,\omega_0}|$ multiplied by all possible pruned branches k and all possible re-grafts at all levels $\sum_{j=1}^{k} j = k(k+1)/2$. Therefore,

$$\mathcal{A}_{n,k,\omega_0} = \frac{k^2(k+1)}{2}|\mathcal{L}_{n,\omega_0}| = \frac{k^2(k+1)n!(n-2)!}{2^{n-1}(1+\delta_{2\omega_0,n})}\,. \tag{S8}$$

The Ω-changing terms of $\mathcal{A}_{n,k,\omega_0,\omega,\mathrm{R}}$ can be computed by averaging over the number of topologically in-equivalent re-graft events y at level j and summing over all levels. We consider only the events where pruning and re-grafting occur in different subtrees of the root, thus potentially changing Ω. The number of ARGs for $\omega \neq \omega_0$ is given by

$$\mathcal{A}_{n,k,\omega_0,\omega,\mathrm{R}} = |\mathcal{L}_{n,\omega_0}|k\sum_{x=2}^{\min(k-2,\omega_0)} P(x|\omega_0,k,n)\left[\frac{x}{k}P(\omega_0-\omega|x,\omega_0)\sum_{j=2}^{k}\sum_{y=1}^{\min(j-1,k-x)} yP(y|k-x,j,k) + \right.$$
$$\left. + \frac{k-x}{k}\frac{P(\omega-\omega_0|k-x,n-\omega_0)+P(n-\omega_0-\omega|k-x,n-\omega_0)}{1+\delta_{2\omega,n}}\sum_{j=2}^{k}\sum_{y=1}^{\min(j-1,x)} yP(y|x,j,k)\right] \tag{S9}$$

Finally, taking the ratio of equation (S9) and $\mathcal{A}_{n,k,\omega_0}$ and averaging, we obtain equation (40).

The above number $\mathcal{A}_{n,k,\omega_0,\omega,R}$ of ARGs with a single recombination event of type R and root imbalances ω_0 and ω can be obtained as follows. We consider all possible trees with ω_0 and a recombination event at level k, that is, $k\mathcal{L}_{n,\omega_0}$ possibilities, then we multiply by the average number of possible prune and regraft events at levels k and j respectively, with $j \leq k$, then summing over all j, k. We use the notation of Figure S4. Pruning occurs at level k and we denote by i the size of the pruned branch. Assume first that the pruning occurs in the left, smaller subtree of the root of size ω_0. The probability that this occurs is x/k and the probability that the pruned branch has size i is $P(i|x,\omega_0)$ as discussed before. The number of possible regraft events at level j is y, that is however a random variable whose probability distribution is $P(y|k-x,j,k)$ defined in equation (35), since it is defined by the same process restricted to the upper part of the tree above level k. We average over y and sum over j, then we average over the probability distribution of x. Finally, the value of ω should be $\omega_0 - i$ since $\omega_0 - i < n/2$, therefore $i = \omega_0 - \omega$. Putting together this contribution and the two similar contributions from the pruning events on the right, larger subtree of the root, we obtain the result (S9).

Moments

Here we report the moments involved in the computation of the probabilities $P^{(c)}$ using the Taylor expansion (eq 19). The moments of the waiting times are

$$\mathrm{E}\left(t_k\right) = \frac{1}{k(k-1)} \qquad \frac{\mathrm{Cov}(t_k, t_j)}{\mathrm{E}(t_j)} = \delta_{kj}\frac{1}{k(k-1)} \tag{S10}$$

$$\mathrm{E}\left(e^{-2dt_d}\right) = \frac{d-1}{d+1} \qquad \frac{\mathrm{Cov}(t_k, e^{-2dt_d})}{\mathrm{E}(e^{-2dt_d})} = \delta_{kd}\frac{-2}{d(d-1)(d+1)} \tag{S11}$$

$$\mathrm{E}\left(\frac{1-e^{-2kt_k}}{2k}\right) = \frac{1}{k(k+1)} \qquad \frac{\mathrm{Cov}(t_k, \frac{1-e^{-2jt_j}}{2j})}{\mathrm{E}(\frac{1-e^{-2jt_j}}{2j})} = \delta_{kj}\frac{1}{k(k+1)} \tag{S12}$$
